# An Overview of Altered Pathways Associated with Sensitivity to Platinum-Based Chemotherapy in Neuroendocrine Tumors: Strengths and Prospects

**DOI:** 10.3390/ijms25168568

**Published:** 2024-08-06

**Authors:** Erika Stefàno, Federica De Castro, Antonella Ciccarese, Antonella Muscella, Santo Marsigliante, Michele Benedetti, Francesco Paolo Fanizzi

**Affiliations:** Department of Biological and Environmental Sciences and Technologies (DiSTeBA), University of Salento, Via Monteroni, I-73100 Lecce, Italy; erika.stefano@unisalento.it (E.S.); federica.decastro@unisalento.it (F.D.C.); antonella.ciccarese@unisalento.it (A.C.); antonella.muscella@unisalento.it (A.M.); santo.marsigliante@unisalento.it (S.M.); fp.fanizzi@unisalento.it (F.P.F.)

**Keywords:** neuroendocrine neoplasms, well-differentiated neuroendocrine tumors, poorly differentiated neuroendocrine carcinomas, platinum-based chemotherapy, response to platinum chemotherapy, molecular pathways mutations, DNA repair pathways mutations

## Abstract

Neuroendocrine neoplasms (NENs) are a diverse group of malignancies with a shared phenotype but varying prognosis and response to current treatments. Based on their morphological features and rate of proliferation, NENs can be classified into two main groups with a distinct clinical behavior and response to treatment: (i) well-differentiated neuroendocrine tumors (NETs) or carcinoids (with a low proliferation rate), and (ii) poorly differentiated small- or large-cell neuroendocrine carcinomas (NECs) (with a high proliferation rate). For certain NENs (such as pancreatic tumors, higher-grade tumors, and those with DNA damage repair defects), chemotherapy is the main therapeutic approach. Among the different chemotherapic agents, cisplatin and carboplatin, in combination with etoposide, have shown the greatest efficacy in treating NECs compared to NETs. The cytotoxic effects of cisplatin and carboplatin are primarily due to their binding to DNA, which interferes with normal DNA transcription and/or replication. Consistent with this, NECs, which often have mutations in pathways involved in DNA repair (such as Rb, MDM2, BRCA, and PTEN), have a high response to platinum-based chemotherapy. Identifying mutations that affect molecular pathways involved in the initiation and progression of NENs can be crucial in predicting the response to platinum chemotherapy. This review aims to highlight targetable mutations that could serve as predictors of therapeutic response to platinum-based chemotherapy in NENs.

## 1. Introduction

Neuroendocrine neoplasms (NENs) are a diverse group of malignancies that originate from neuroendocrine (NE) cells, which are characterized by both “neuro” and “endocrine” properties. These cells release hormones into the bloodstream in response to nervous system stimulation [1]. The classification of neuroendocrine neoplasms is based on the primary site of origin, proliferation index (Ki-67), and symptoms caused by the production of biologically active amines (functioning and non-functioning NENs) [2].

**Figure 1:**
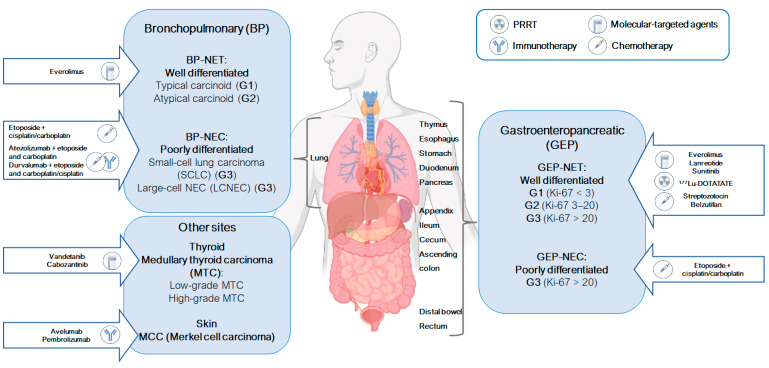
Classification and treatment options for neuroendocrine neoplasms (NENs). Well-differentiated neuroendocrine tumors (NETs) are categorized as G1, G2 and G3, while poorly differentiated neuroendocrine carcinomas (NECs) are classified as G3. Resectable tumors are best treated with surgery, while systemic therapeutic options such as somatostatin analogs (SSAs, e.g., lanreotide), mTOR inhibitors (e.g., everolimus), tyrosine kinase inhibitors (TKIs, e.g., sunitinib, vandetanib, cabozantinib), immunotherapy (e.g., avelumab, pembrolizumab), chemotherapy (e.g., streptozotocin, etoposide with cisplatin/carboplatin, belzutifan), and peptide receptor radionuclide therapy (PRRT) with ^177^Lu-DOTATATE (^177^Lutetium-[DOTA(0),Tyr(3)]octreotate) are available for inoperable or metastatic disease.

Since NE cells are found throughout the body, neuroendocrine tumors can arise in various tissues, including the skin, nervous system, respiratory tract, gastrointestinal tract, larynx, thyroid, breast, and urogenital system. The neuroendocrine system includes endocrine glands (such as the parathyroid, pituitary, and adrenal glands), as well as endocrine islet tissue embedded within glandular tissue (such as the thyroid or pancreas) and scattered cells in the exocrine parenchyma (such as the endocrine cells of the digestive and respiratory tracts in the diffuse endocrine system) [1,3]. The most common primary tumor sites for neuroendocrine neoplasms are the gastroenteropancreatic (GEP) and bronchopulmonary (BP) tracts, although they can develop in any organ or system in the human body, with similar features due to their neuroendocrine nature (Figure 1).

Neuroendocrine neoplasms (NENs) can also be classified based on the specific hormones they secrete [1,4,5,6,7,8]. In fact, they can oversecrete bioactive substances that regulate certain body functions, which results in a clinical syndrome known as carcinoid syndrome. Tumors associated with this syndrome are currently defined as “carcinoids”, which are well established and distinct clinical entities [9]. The degree of biological aggressiveness and response to therapies of NENs [7,10] is influenced by their secretory properties and syndromes of uncontrolled hormone hypersecretion (such as Cushing, Verner–Morrison, Zollinger–Ellison, and other eponymic syndromes). Therefore, the challenging management of NENs is due to their heterogeneous clinical presentations and varying degrees of aggressiveness [11].

In order to standardize the nomenclature of NENs, the 2015 World Health Organization (WHO) proposed a universal definition system based on mitotic count and/or Ki-67 index and/or the presence of necrosis, classifying NENs into three tiers (grades 1–3). Based on this concept, NENs are divided into well-differentiated neuroendocrine tumors (NETs) (G1, G2 and G3 grade) and poorly differentiated neuroendocrine carcinomas (NECs), which are high-grade neoplasms (G3) (Figure 1) [7,10]. NETs and NECs have different risk factors, hereditary predispositions, relationships to non-NEN, and genetic factors (for example, NECs are most frequently characterized by p53 and Rb gene alterations compared to NETs) [7,10]. Furthermore, NECs should be distinguished from carcinoids: both are composed of chromogranin-positive neuroendocrine cells but, while carcinoids are low-grade malignancies, NECs are highly aggressive malignancies [12]. These types of neoplasms can also be composed of different combinations of NENs (NET or NEC) and non-NENs, which are referred to as mixed NENs/non-NENs (MiNENs) [13].

Neuroendocrine neoplasms can also be classified by identifying conventional biomarkers of NE lineage and differentiation, which are useful in distinguishing G3 NETs from NECs, epithelial from non-epithelial NENs, and so on. Some examples of biochemical markers for NENs include chromogranin A (CgA), pancreatic polypeptide (PP), human chorionic gonadotropin (HCG), alpha-fetoprotein (AFP), neuron specific enolase (NSE) [10,14], insulinoma-associated protein-1 (INSM-1), synaptophysin (SYN), and somatostatin receptors (SSTRs). Transcription factors (e.g., thyroid transcription factor-1, TTF-1; Islet 1, Isl-1; paired box 8, PAX 8), enzymes, keratins, and hormones can also be useful in functional and structural correlation. For example, insulinomas, glucagonomas, gastrinomas, somatostatinomas, and vipomas are named after the hormones they produce (insulin, glucagon, gastrin, somatostatin, and vasoactive intestinal peptide (VIP), respectively) [7,14,15,16].

Generally, NENs are sporadic, but they may also arise due to hereditary syndromes that predispose individuals to the onset of neuroendocrine neoplasms, such as multiple endocrine neoplasia type 1 and 2 (MEN-1 and MEN-2 hereditary cancer syndromes), von Hippel–Lindau (VHL) syndrome, neurofibromatosis, and tuberous sclerosis [1,17]. NENs can also be associated with mutations in different pathway genes, as rearranged during transfection (RET) proto-oncogene, mTOR (mammalian target of rapamycin), and VEGF (vascular endothelial growth factor)/VEGF-receptor pathway genes [2,18]. The PI3K-Akt-mTOR (phosphatidylinositol 3-kinase-Akt-mammalian target of rapamycin) pathway plays an important role in NENs. In fact, mTOR inhibitors (rapamycin analogs) have been approved by the FDA (Food and Drug Administration) for the treatment of advanced pancreatic NETs [19]. The treatment of NENs generally involves a variety of therapeutic approaches (surgery, radiotherapy, immunotherapy, molecular-targeted agents, and chemotherapy), depending on the tumor grade and differentiation [7,20] (Figure 1).

**Figure 2:**
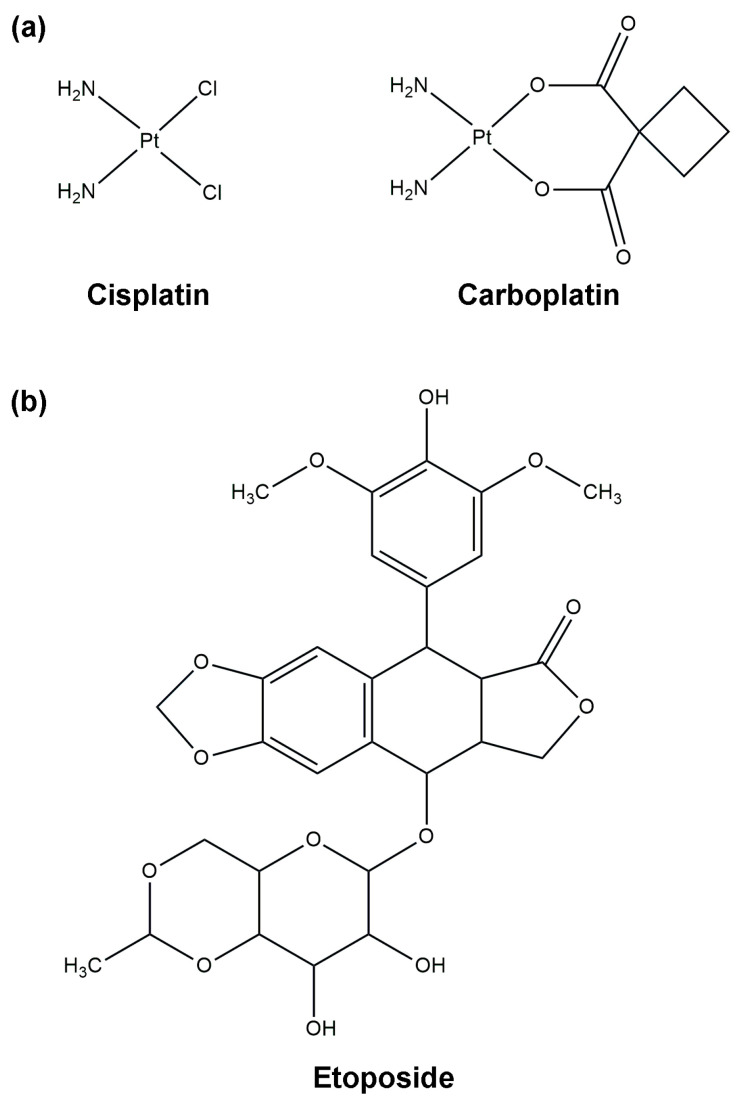
Chemical structures of cisplatin, carboplatin and etoposide. Cisplatin or carboplatin (**a**), in combination with etoposide (**b**), are the cornerstone of chemotherapy treatment for various types of cancers, including poorly differentiated or undifferentiated, high-grade neuroendocrine tumors (NETs), small-cell lung cancer (SCLC), and large-cell neuroendocrine carcinoma (LCNEC).

Currently, platinum-based chemotherapy is considered a first-line palliative treatment for various types of tumors, including NENs [21,22,23]. For poorly differentiated NECs, such as gastroenteropancreatic neuroendocrine cancers (GEP-NECs) and bronchopulmonary neuroendocrine cancers (BP-NECs), the most effective therapy involves the use of cisplatin or carboplatin in combination with etoposide (Figure 2) [21]. Despite the widespread use of platinum agents in various tumor types, their mechanism of action in NE tumor cells remains unclear.

This review stems from the need to better understand the predictive and prognostic features of NENs. These features remain uncertain, making it crucial to identify the major molecular genetic alterations in each type of tumor. Establishing correlations between specific genetic abnormalities involved in tumorigenesis and metastasis could reveal potential targets for cancer therapy. Due to the heterogeneity of NENs, there is a significant need to re-evaluate chemotherapeutic approaches, focusing on combined and personalized therapies that offer greater selectivity and effectiveness. The review incorporates recent and relevant studies in the field, providing a comprehensive analysis of current knowledge and developments in NENs and responses to platinum-based chemotherapy. This analysis considers data and research findings up to the year 2024 and discusses the role of altered molecular pathways in NENs that contribute to sensitivity to platinum drugs. These genomic alterations are typically involved in tumor initiation and progression and serve as biomarkers for predicting therapeutic outcomes. Therefore, the purpose of this work is to identify the main affected signaling pathways in NENs and their relationship to response to platinum chemotherapy.

### 1.1. Most Common Neuroendocrine Neoplasms

#### 1.1.1. Gastroenteropancreatic Neuroendocrine Neoplasms (GEP-NENs)

Gastroenteropancreatic neuroendocrine neoplasms (GEP-NENs) affect organs of the gastrointestinal tract. Approximately 30% of GEP-NENs are hormonally active and can produce and secrete peptides and neuroamines causing specific clinical syndromes. Poorly differentiated neuroendocrine carcinomas (NECs) account for 10–20% of malignant GEP-NENs and are primarily found in the esophagus, pancreas, ampulla of Vater, large bowel, and rarely in the ileum (Figure 1) [24].

Their clinical features mainly depend on the primary site of the tumor and its functionality [25]. Based on the primary tumor site, GEP-NENs are divided into two sub-categories: carcinoid tumors of the luminal gastrointestinal (GI) tract and pancreatic (P) neoplasms [1,6]. Among GEP neoplasms, pancreatic NETs (P-NETs) account for approximately 1% of pancreatic cancers. An estimated 40–91% of P-NETs are non-functioning, while the others manifest evident hormonal symptoms (e.g., insulinoma, gastrinoma, glucagonoma, vipoma, somatostatinoma) [26,27]. GI-NETs and P-NETs may have similar histological features but variable clinical behavior and biology. P-NETs have a relatively worse prognosis than GI-NETs and respond differently to therapies. Several agents have shown higher response rates in P-NETs compared to GI-NETs [28,29,30].

#### 1.1.2. Bronchopulmonary Neuroendocrine Neoplasms (BP-NENs)

Similar to GEP-NENs, bronchopulmonary neuroendocrine tumors (BP-NENs) are classified based on morphology and/or mitotic count. The World Health Organization (WHO) classification (2015) groups both, lung and thymic neuroendocrine tumors (NETs) (referred to as neoplasms in the digestive WHO classification) into one category, subdivided into two main groups: (a) BP-NETs, which include low-grade typical carcinoid (TC) (0–1 mitoses per 2 mm^2^) and intermediate-grade atypical carcinoid (AC) (2–10 mitoses per 2 mm^2^), and (b) BP-NECs, which consist of large-cell neuroendocrine carcinoma (LCNEC) and small-cell lung carcinoma (SCLC) (≥11 mitoses per 2 mm^2^) (Figure 1) [6,31].

The accurate identification and differentiation of AC from TC or NECs (LCNEC and SCLC) is crucial for determining treatment options and prognosis [32]. Currently, the standard treatment for patients with SCLC involves platinum-based chemotherapy combined with an immune checkpoint inhibitor (ICI) such as atezolizumab or durvalumab [29] (Figure 1). The histological features of LCNEC can overlap with non-small-cell lung cancer (NSCLC) and, in some cases, SCLC, making a histological diagnosis challenging and requiring the establishment of an optimal systemic treatment. On the other hand, chemotherapy for SCLC is considered the most appropriate treatment (Figure 1) [33,34].

## 2. Approved Therapeutic Options for Neuroendocrine Neoplasms

As previously discussed, neuroendocrine neoplasms (NENs) encompass a wide range of tumors with varying biological and clinical features. As a result, there has been a noticeable expansion in therapeutic options for NENs in recent years, particularly for well-differentiated NETs [6]. NENs can be differentiated based on laboratory tests (secretory vs. non-secretory), clinical symptoms (functioning vs. non-functioning), morphological features (growth patterns of cancer cells, mitotic counts, Ki-67 index, necrosis, expression of somatostatin receptors, SSTRs), and the degree of cellular differentiation. Due to the significant differences in clinical behavior between G1 and G2 NETs compared to G3 NETs, treatment approaches vary between these two malignancies [35]. Additionally, G3 NETs have distinct biological features compared to poorly differentiated G3 neuroendocrine carcinomas (NECs) [28,36,37,38].

The identification of altered pathways involved in the pathogenesis of NENs has led to the development of specific therapies [39]. Surgery is the preferred option for resectable tumors, while for inoperable or metastatic disease, therapeutic options include radiation therapy, somatostatin analogs (SSAs), mTOR inhibitors, receptor tyrosine kinase receptors inhibitors (TKIs), chemotherapy, peptide receptor radionuclide therapy (PRRT), and targeted therapy (Figure 1) [40,41].

In recent years, molecular-targeted therapies have emerged as a treatment approach for advanced neuroendocrine tumors (NETs) [42]. Among these therapies, somatostatin analogs (SSAs) have been shown to delay tumor progression and decrease hormone overproduction by interacting with somatostatin receptors (SSTRs), which are often overexpressed in NETs [43]. Somatostatin (SST) plays a role in regulating cell growth and hormone secretion, making SSTRs a potential target for treating neuroendocrine neoplasms (NENs) [43]. The first synthetic SSA to be approved by the FDA was octreotide, an octapeptide available in both conventional and long-acting release (LAR) injections (approved in 1988 and 1998, respectively). Lanreotide was initially approved for treating acromegaly but has since been approved for treating unresectable, well- or moderately differentiated, locally advanced, or metastatic gastroenteropancreatic (GEP) NETs (in 2014) and carcinoid syndrome (in 2017).

Another type of targeted therapy is represented by everolimus and sunitinib, which were approved by the FDA in 2011. Everolimus is an mTOR inhibitor that plays a role in the tumorigenesis and progression of NENs, while sunitinib is a multi-targeted tyrosine kinase inhibitor (TKI) that blocks the activation of VEGFRs 1–3 (vascular endothelial growth factor receptor 1–3), PDGFR-α and -β (platelet-derived growth factors-α and -β), KIT (stem-cell growth factor receptor), FLT3 (fms-related tyrosine kinase 3), RET and CSF1R (colony-stimulating factor receptor 1), resulting in antiangiogenic and antitumor activity against a broad range of neoplasms. The FDA has approved these targeted agents for managing advanced GEP-NETs: everolimus for both primary gastrointestinal NETs (GI-NETs) and pancreatic NETs (P-NETs) and sunitinib for primary P-NETs [44,45,46,47]. Vandetanib and cabozantinib are TKIs currently used to treat patients with unresectable, progressive, and symptomatic medullary thyroid carcinoma (MTC) [48,49,50] (Figure 1).

Peptide receptor radionuclide therapy (PRRT) is a treatment option for patients with progressive somatostatin receptor (SSTR)-positive neuroendocrine tumors (NETs) [43,51]. PRRT involves attaching a radioisotope to a chelating molecule, which is then linked to a peptide that targets SSTRs on the surface of cancer cells. This allows for the precise delivery of radiation to the tumor. In the case of advanced NETs, the peptide used can be a somatostatin analog (SSA) or antagonist that binds to SSTRs. In 2018, the FDA approved the radiopharmaceutical lutetium (^177^Lu) for the treatment of patients with SSTR-positive gastroenteropancreatic NETs (GEP-NETs) (Figure 1) [52].

Immunotherapy is another treatment option for neuroendocrine neoplasms (NENs), that has achieved significant success in treating small-cell lung cancer (SCLC) and Merkel cell carcinoma (MCC) [53]. CTLA-4 (cytotoxic T-lymphocyte antigen 4), PD-1 (programmed death-1) and PD-L1 (programmed death-1 ligand) are involved in cancer cells evading immune surveillance. Immune checkpoint inhibitors (ICIs) are antibodies that target these molecules and have proven effective in various cancers, included NENs. Avelumab and pembrolizumab are the only two FDA-approved ICIs for treating metastatic MCC, a rare and aggressive neuroendocrine tumor of the skin (Figure 1) [53,54,55].

Cytotoxic chemotherapy is the standard treatment for patients with poorly differentiated neuroendocrine carcinomas (NECs), but its role in patients with well-differentiated neuroendocrine tumors (NETs) is not well defined [56]. Streptozotocin (STZ) is an alkylating agent that was the first drug to show efficacy in NETs.

It was approved in 1982 for treating pancreatic NETs. Subsequent studies have shown that STZ in combination with 5-fluorouracil (5-FU) and/or doxorubicin is effective for treating well-differentiated pancreatic NETs (P-NETs) [57,58,59,60]. In 2021, the FDA approved belzutifan, a hypoxia-inducible factor inhibitor (HIF-2α), for certain types of cancers, including P-NETs associated with von Hippel-Lindau disease [61]. Recently, the combination therapy of capecitabine and temozolomide (CAPTEM) showed improved anticancer activity in metastatic P-NETs [62] (Figure 1).

**Figure 3:**
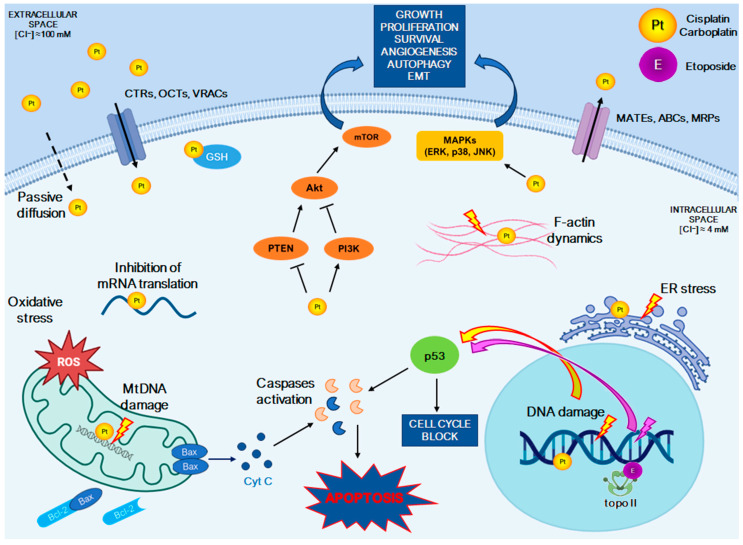
General accepted mechanism of action of cisplatin/carboplatin etoposide combined therapy essentially showing the platinum drugs contribution. Cisplatin and carboplatin enter cells through passive diffusion and various transport proteins, including copper transporters (CTR1–2), organic cation transporters (OCT1–3), and LRRC8 volume-regulated anion channels (VRACs). Some of these transporters are associated with the export of platinum drugs and drug resistance, such as P-type copper-transporting ATPases (ATP7A and ATP7B), multidrug extrusion transporters (MATE 1–3), and ATP-binding cassette (ABC) transporters (MRP1–2). Once inside the cell, cisplatin and carboplatin undergo hydrolysis of chloro-ligand(s) and 1,1-cyclobutanedicarboxylate, respectively, resulting in a positively charged form. This allows the platinum compounds to interact with nucleophilic molecules within the cell, including DNA, RNA, and proteins, leading to the formation of platinum adducts. The formation of DNA adducts inhibits the cell cycle and impairs DNA repair, ultimately causing DNA damage and p53 activation, which induces apoptosis. Additionally, cisplatin and carboplatin induce intrinsic apoptosis by increasing mitochondrial ROS generation and activating pro-apoptotic proteins, such as Bax, which promotes the release of cytochrome C (Cyt-C) and the subsequent activation of caspases. Etoposide is a topoisomerase II inhibitor, which is considered a major anticancer mechanism of this drug. The combined actions of cisplatin or carboplatin and etoposide enhance DNA damage and promote cancer cell death.

Among the chemotherapeutic agents mentioned, cisplatin/carboplatin-based therapy is the first-line regimen (Figure 1 and Figure 2). However, not all platinum compounds have the same activity in neuroendocrine neoplasms [63]. Cisplatin with etoposide-based therapy appears to be more effective in G3 NET (with Ki-67 > 50%) and metastatic NECs (Figure 3) [21]. On the other hand, NEC patients with Ki-67 < 55% are less responsive to platinum-based chemotherapy but have a longer survival than those with a higher Ki-67.

Of the FDA-approved platinum compounds, oxaliplatin has shown significant clinical results in patients with well differentiated NETs, rather than cisplatin or carboplatin. The most commonly tested oxaliplatin combination regimens in patients with NETs are fluorouracil plus oxaliplatin (FOLFOX) or capecitabine plus oxaliplatin (CAPOX) [56]. Oxaliplatin has also demonstrated significant anticancer activity in G2 NETs and carcinoids, but there is currently no universally accepted standard chemotherapy for these tumors [40,63].

Significant effects have been observed when platinum-based drugs are combined with molecular-targeted therapy, immunotherapy, or other cytotoxic agents [29,53,64,65]. In 2019, the FDA approved atezolizumab in combination with etoposide and carboplatin [65] and, in 2020, durvalumab (an IgG1 kappa anti-PD-L1 monoclonal human antibody) in combination with etoposide and carboplatin/cisplatin as first-line treatments for patients with extensive-stage (ES) SCLC (ES-SCLC) (Figure 1) [53].

## 3. Platinum-Based Chemotherapy

Platinum-based antitumor drugs are a successful class of chemotherapy agents [66,67,68]. Cisplatin was the first metal-based anticancer drug introduced into clinical use in 1978 for several types of solid tumors [69]. However, its use as an anticancer drug is limited due to side effects such as neurotoxicity, nephrotoxicity, hepatotoxicity, and myelosuppression [22]. To overcome the high toxicity and chemoresistance associated with cisplatin-based therapy, a very high number of new platinum complexes have been synthesized and tested for antitumor activity [67,68,70,71,72,73,74,75,76,77,78]. However, only its analogs carboplatin and oxaliplatin have been approved by the FDA as chemotherapeutic drugs. Other platinum agents, such as nedaplatin, lobaplatin, and heptaplatin, have been approved only in Japan, China, and South Korea, respectively [66,67,68].

Currently, cisplatin and its derivatives are used to treat lymphomas, lung, colon, ovary, testicular, bladder, cervical, and other types of cancer, including neuroendocrine cancers [22,40]. These drugs induce cytotoxic effects by binding to DNA, interfering with its normal transcription and/or replication (Figure 3) [68,70,72,79]. In order to enhance and optimize the antitumor activity of cisplatin analogs, new coordination compounds are under evaluation as alternative platinum drugs. In recent years, platinum-based anticancer complexes have made significant progress in cancer therapy. New anticancer molecules have been synthesized or developed by modifying existing platinum drugs and attempting to mimic the mechanism of action of cisplatin [67,68,70,71,72,73,75,76,77,80,81,82,83,84,85,86,87,88]. However, no new platinum complexes have been approved for cancer therapy, neuroendocrine neoplasms included.

### General Mechanism of Action of Cisplatin and Carboplatin

Cisplatin (*cis*-diamminedichloroplatinum(II)) and carboplatin (*cis*-diammine(1,1-cyclobutanedicarboxylato)platinum(II)), in combination with etoposide (Figure 2), represent the current standard first-line chemotherapy for various types of tumors, including neuroendocrine cancers. By 1979, pre-clinical data had shown synergistic effects of the combination of cisplatin and etoposide [89,90].

Platinum-based anticancer drugs can generally enter cells through passive diffusion and also using various cell membrane transport proteins, such as copper transporter 1 and 2 (CTR1 and CTR2), P-type copper-transporting ATPases (ATP7A and ATP7B), the organic cation transporter 2 (OCT2), the multidrug extrusion transporter 1 (MATE1), and LRRC8 volume-regulated anion channels (VRACs) (Figure 3) [91,92,93]. In the extracellular matrix, the concentration of chloride ions is higher (~100 mM) than in intracellular environment (~4 mM). As a result, after entering the cell, cisplatin undergoes an activation step where chloro-ligands are replaced by water molecules or other molecules containing sulfhydryl groups. This “aquation” of cisplatin promotes the formation of mono- and di-aquo species, such as *cis*-[Pt(NH_3_)_2_Cl(OH_2_)]^+^, *cis*-[Pt(NH_3_)_2_(OH)(OH_2_)]^+^ and *cis*-[Pt(NH_3_)_2_(OH_2_)_2_]^2+^ [83]. Due to the chelation of the leaving ligand, carboplatin and oxaliplatin are more stable and their activation is allowed by nucleophiles containing sulfhydryl groups, such as glutathione (GSH), aspartic acid, and other molecules [83,94,95]. The cytotoxicity induced by cisplatin and carboplatin is primarily due to their binding to DNA, through the formation of mono- and bis-adducts, producing intra- and inter-strand cross-links with DNA. However, it was estimated that only about 1% of intracellular cisplatin interacts with nuclear DNA [96]. It has been demonstrated that in the cytoplasm, platinum drugs interact with other biomolecules, such as cellular proteins, membrane phospholipids, and RNA [96], and induce cytotoxicity through the acidification of the cytoplasm, ER stress, the inhibition of RNA transcription and translation, the inhibition of important oncogenic proteins, and a decrease in metabolic plasticity of cancer cells [88,97,98]. The general accepted mechanism of action of cisplatin/carboplatin etoposide combined therapy essentially showing the platinum drugs contribution is reported in Figure 3.

In the nucleus, the diamineplatinum(II) units are coordinated by the N7 of purine bases, leading to the inhibition of replication and/or transcription, DNA damage, interference with DNA repair mechanisms, and ultimately, cell cycle arrest at S, G1 or G2-M and the induction of apoptosis/necrosis in cancer cells [67,83,99,100,101]. The interaction of cisplatin and carboplatin with DNA activates several signal transduction pathways, including those involving ATR, p53, p73, MAPKs (such as ERK, p38, and JNK), and PI3K/Akt, which ultimately result in the induction of apoptosis through both intrinsic and extrinsic pathways [68,102,103,104,105,106,107]. The formation of cisplatin/carboplatin adducts to DNA also activates the tumor suppressor p53, which can activate genes involved in cell cycle progression, DNA repair, and apoptosis [86,105,107]. The p53 protein can also activate genes of the Bcl family, which include pro-apoptotic (e.g., Bax and Bak) and anti-apoptotic (e.g., Bcl-2) factors (Figure 3) [108].

The combination of cisplatin or carboplatin drugs with etoposide improves DNA damage and induces cancer cell death [83,109]. Etoposide, a derivative of podophyllotoxin, was first synthesized in 1966 and approved for cancer therapy by the FDA in 1983. It targets DNA topoisomerase II (topo II) activity, inhibiting the faithful rejoining of DNA breaks and affecting various aspects of cell metabolism. Specifically, it causes topo II-linked DNA double- or single-strand breaks by inhibiting the rejoining of cleaved DNA [109]. Studies have shown that etoposide-induced DNA damage activates p53, leading to cell death [110,111]. In the apoptotic cascade, the activation of DNA-PK (DNA-dependent protein kinase) is crucial as it links the recognition of DNA damage to downstream signaling events. The activation of p53 by etoposide results in the upregulation of the pro-apoptotic protein Bax and the release of cytochrome c (Cyt C) (Figure 3) [112,113]. Robertson and colleagues hypothesized that etoposide-induced DNA damage leads to the activation of caspase-2, which acts as an intermediary in the induction of the mitochondrial apoptotic pathway [113,114]. On the other hand, while it is well known that etoposide triggers apoptotic pathways, recent findings also suggest its involvement in autophagic pathways [115,116]. Specifically, etoposide-induced autophagy seems linked to the activation of AMPK (AMP-activated protein kinase) [115]. Indeed, etoposide could induce an autophagy-associated surge in ATP, which contributes to cell survival and drug resistance [117].

## 4. Altered Pathways in NENs and Platinum-Based Chemotherapy Sensitivity

**Figure 4:**
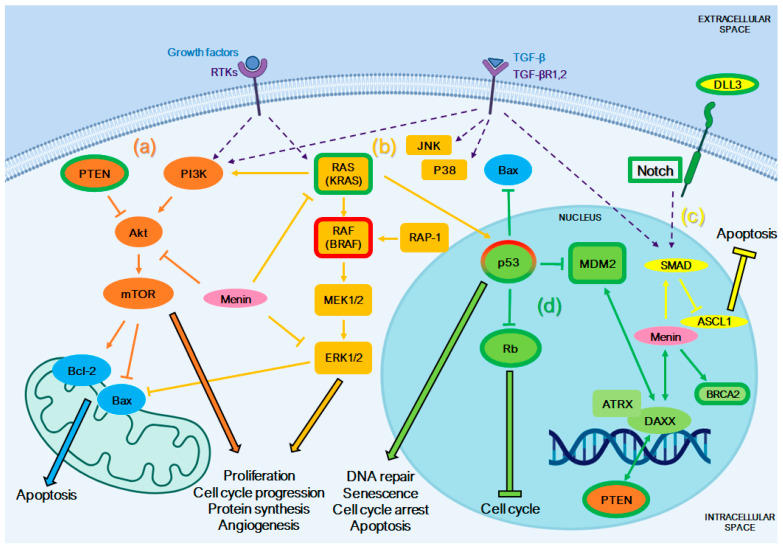
Signal transduction pathways influencing the efficacy of platinum-based chemotherapy in neuroendocrine neoplasms (NENs). Genetic alterations or abnormal expression of pathway members, indicated in green and red respectively, have been linked to increased or decreased sensitivity to platinum-based therapy. Growth factors (EGF, FGF, IGF, TGF-α, etc.) can bind to tyrosine kinase receptors (RTKs) and activate the (a) PTEN/PI3K/Akt and (b) RAS/RAF/MAPK pathways, resulting in the transcription of genes associated with cell proliferation, invasion, and metastasis. Mutations in (a) PTEN and (b) KRAS have been associated with heightened cytotoxic effects following platinum drug administration, while (b) BRAF mutations have been linked to limited response to chemotherapy. TGF-β can interact with TGF-β receptors (TGF-βR1,2) and activate the (a) PTEN/PI3K/Akt, (b) MAPK, and (c) SMAD pathways. In NE cells, the disruption of TGF-β signaling leads to an increased expression of (c) ASCL1, which in turn protects cancer cells from apoptosis. Additionally, (c) Notch signaling can activate ASCL1 through SMAD-mediated activation. The low expression of both Notch1 and DLL3 has been associated with better prognosis and increased sensitivity to platinum chemotherapy. Mutation in DNA repair-related genes can also impact response to platinum drugs. (d) The role of p53 in chemotherapy response is still unclear, with alterations in this gene being linked to both negative and neutral effects. However, the overexpression of the p53-negative regulator MDM2 in NENs has been shown to improve response to platinum-based therapy. Mutations of Rb and BRCA2 have also been associated with improved response to cisplatin chemotherapy. (a–c) Menin is implicated in the regulation of several of the aforementioned pathways.

The anticancer activity of cisplatin and carboplatin was extensively studied in vitro and in vivo by various research groups for several types of tumors (Figure 3) [98,118]. Despite their widespread use in treating different types of neoplasms, platinum-based chemotherapy is often ineffective in treating NENs, and the mechanism of action in NE tumor cells remains unclear. Several pathways have been found to be altered in cisplatin/carboplatin sensitivity and are discussed below (Figure 4).

### 4.1. PTEN/PI3K/Akt/mTOR Pathway

The PI3K/Akt/mTOR pathway plays a crucial role in the development, progression, and angiogenesis of NENs, making it a promising target for treatment [119,120,121,122,123]. Akt, a major downstream regulator of PI3K, promotes cell proliferation by deactivating pro-apoptotic genes, such as caspase and Bcl-2 family members [107]. Moreover, mTOR is essential for the activation of the autophagic process, a key homoeostatic machinery of cellular self-degradation. Interestingly, across different tumor types, autophagy exhibits promoting or inhibitory effects to tumorigenesis by favoring resistance to anticancer treatments or inducing tumor cell cycle arrest, respectively [27,124,125,126]. The autophagic activity or the expression of autophagy-associated genes to influence survival in NENs has not been investigated yet, although it seems that a lower expression level of autophagic genes is associated with a metastatic stage [126].

Since the mTOR pathway is consistently activated in NETs, the development of mTOR inhibitors has provided a new therapeutic option for these tumors (Figure 1 and Table S1) [19,33,37,38,127,128,129,130,131,132,133,134,135,136,137,138,139,140,141,142,143,144,145,146,147,148,149,150,151,152,153,154,155,156,157,158,159,160,161,162,163,164,165,166,167,168,169,170,171]. In fact, the PI3K/Akt/mTOR pathway plays an important role in tumorigenesis and the tumor progression of NENs [122]. It was observed that the PI3K/Akt/mTOR axis can cause resistance to cisplatin [172] and carboplatin [173] treatment. Conversely, the inhibition of Akt/mTOR can promoted cisplatin-induced apoptosis in resistant cells [174].

Despite these advancements, there is still much to learn about how platinum drugs interact with NENs, particularly in patients with an abnormal expression of PI3K/Akt/mTOR components (Figure 4). In this regard, we summarize below some studies that have investigated the relationships between the PTEN/PI3K/Akt/mTOR signaling system and the differential sensitivity to platinum-based chemotherapy.

PTEN (phosphatase and tensin homolog) is a tumor suppressor protein that negatively regulates the PI3K/Akt/mTOR signaling axis [122] and stimulates various DNA repair pathways, including homologous recombination (HR), non-homologous end joining (NHEJ), and nucleotide excision repair (NER). Its absence can sensitize cancer cells to DNA-damaging agents, including platinum drugs (Figure 3 and Figure 4) [128,129,175,176,177]. In NECs, the loss or reduced expression of PTEN has been associated with rapid tumor growth, metastasis, and poor survival (Table S1) [37,129,130,178,179]. Omura and colleagues observed that PTEN loss or downregulation was linked to a better response to platinum drugs. They reported the first case of a patient with advanced castration-resistant prostate NEC who showed a significant response to platinum-based chemotherapy and had mutations in both BRCA2 and PTEN (Table 1) [177]. Nuclear PTEN promotes genomic stability and DNA repair through the upregulation of RAD51, a key protein involved in double-strand break repair. Cytoplasmic PTEN also inhibits the Akt-mediated cytoplasmic sequestration of the checkpoint kinase CHEK1, preventing genomic instability and the accumulation of double-strand breaks [180]. By contrast, the loss of PTEN promotes genomic instability and the accumulation of double-strand break repair in tumor cells, enhancing platinum drugs’ anticancer activity [180,181]. However, the frequency of somatic PTEN alterations in patients with neuroendocrine prostate cancer is unknown because of its rarity and the lack of available genomic analysis in the literature [177].

### 4.2. Mitogen-Activated Protein Kinase (MAPK) Pathway

The MAPK pathway is an important regulator of the survival and proliferation of NENs, activated by various growth factors [105,197,198,199,200]. Platinum drugs can also induce apoptosis through the activation of MAPKs, contributing to tumor regression [105,197,201,202,203]. It has been demonstrated that the MAPK signal transduction pathway is associated with NE differentiation, cell growth, autophagy and metastasis (Table S1) [133,202,204]. Downstream of the growth factor receptors, the ERK cascade is initiated by RAS, which activates RAF and recruits it from the cytosol to the cell membrane. RAF then activates MEK, which in turn phosphorylates and activates ERK (Figure 4) [201]. Mutations in RAS and RAF family members have been linked to different clinical behaviors and responses to chemotherapy in NENs (Table S1).

The KRAS oncogene, a member of the RAS family, has the highest mutation rate among all cancers and is associated with high mortality [183,205]. In NENs, it is rarely mutated [201], but its mutational status appears to be related to different patterns of sensitivity or resistance to platinum drugs [182,183,206]. Tanaka and colleagues showed that patients with P-NEC who had KRAS mutations demonstrated an improved response to platinum-containing therapy [183,205]. Hijioka et al. demonstrated that high-grade P-NETs and P-NECs have distinct clinicopathologic features and that pancreatic NENs (G3) with mutated KRAS had a significantly higher response rate (77%) to platinum-based chemotherapy than those without (23%) [182]. Elvebakke et al. reported no influence of KRAS mutations in treatment efficacy or survival for patients with colon NEC receiving first-line platinum/etoposide chemotherapy. Conversely, BRAF mutations were associated with a limited effect of first-line chemotherapy, although they did not affect progression-free survival or overall survival (Figure 4; Table 1) [184].

### 4.3. Notch/ASCL1 Pathway

Notch (neurogenic locus notch homolog)/ASCL1 (Achaete-scute homolog 1) signaling is known to regulate cellular differentiation, proliferation and survival. However, contradictory findings have shown that Notch can act as both an oncogene and a tumor suppressor, indicating that its role is highly dependent on the specific cellular context. The Notch signaling pathway plays a crucial role in the growth and differentiation of GI-NETs [134], and its low expression has been linked to a better prognosis in SCLC patients (Table S1) [131,207]. Conversely, the overexpression of Notch has been shown to inhibit cell proliferation and to induce apoptosis in NET rather than promoting tumor growth [129,208,209]. This can also lead to the modulation of ASCL1 expression, as ASCL1 levels decrease when Notch signaling is active (Figure 4). ASCL1 is a transcription factor that is essential for the development and neuroendocrine differentiation of pulmonary NE cells, SCLC, thyroid C cells, and adrenal chromaffin cells [123]. Additionally, ASCL1 has been found to promote more aggressive growth of pulmonary adenocarcinoma in vivo and can interact with the Rb-p53 axis in the carcinogenesis of NE lung cancers (Table S1) [38,123,210,211,212,213,214].

Notch-negative and ASCL1-positive NE cells appear to be particularly susceptible to cytotoxic chemotherapy during initial treatment. However, epigenetic mechanisms that induce Notch expression in residual cancer cells may lead to recurrence in patients after repeated chemotherapy [38,207,215,216].

DLL3 (Delta-like protein 3) is a member of the Notch receptor ligand family that inhibits Notch signaling and is considered a predictive marker of sensitivity to platinum-based chemotherapy for LCNEC. Among patients with DLL3-negative LCNEC, platinum-based adjuvant chemotherapy has been shown to significantly improve overall survival and recurrence-free survival (Figure 4). However, patients with DLL3-positive LCNEC do not demonstrate improved response to chemotherapy (Table 1) [185]. In contrast, Tendler et al. found that an abnormal expression of ASCL1 and DLL3 in SCLC did not result in differences in clinical outcome. However, patients with a low expression of Notch-1 had a better prognosis and higher sensitivity to platinum-based chemotherapeutic drugs (Figure 4; Table 1) [131].

### 4.4. Pathways Involved in DNA Repair

Several studies have demonstrated that aberrations in DNA repair genes, including MDM2, RB, BRCA2, and MEN-1 mutations, serve as biomarkers for a heightened response of platinum-based chemotherapy (Figure 4). The combination of platinum drugs and etoposide in NENs results in increased DNA damage, leading to the inhibition of DNA repair and replication [217]. In P-NETs, mutations in PTEN, MEN1, and DAXX/ATRX genes were found to be common. The loss of these tumor suppressors in NENs may render them more susceptible to the cytotoxic effects of platinum-based drugs (Figure 4) [20,135,136,137,218].

The tumor suppressor p53, which is involved in cell cycle progression, DNA repair, and apoptosis, is activated after the formation of platinum drug adducts to DNA [86,105,107]. Some researchers have suggested that the inactivation of p53 and downstream DNA repair-related genes are responsible for platinum resistance mechanisms in NENs [219]. The role of p53 regulation in NENs and its consequences on chemotherapy efficacy are controversial. Mutant p53 proteins commonly lose wild-type function but can also acquire novel functions in promoting metastasis and resistance to platinum drugs and etoposide [220]. In NSCLC with NE differentiation, no significant correlation was found between altered p53 expression and response to platinum-based chemotherapy, although the increased expression of p53 was related to progressive disease following chemotherapy [132]. Similarly, Elvebakken and colleagues observed a limited response to treatment with platinum/etoposide in patients with high-grade GEP-NEN. Moreover, also a significantly better survival was observed in small-cell NEC [189]. In high-grade NENs, which often have alterations in the p53 gene (Table S1), p53 expression was not related to improved chemotherapy response [20,191]. On the other hand, some studies associated TP53 mutations or abnormal p53 protein expression with higher sensitivity to platinum therapy [187,188]. Some discrepancies in these results could be explained by the existence of different *TP53* mutations which can differently modulate p53 accumulation in the cell nuclei. Moreover, the prognostic value of TP53 mutations might depend on co-mutations and tumor type. Finally, mutational analyses are recommended for the evaluation of clinical outcomes in order to bypass discordance between protein p53 measurements and TP53 mutation frequency [189].

Although TP53 gene mutations are rare in NENs [138,219,221], epigenetic and regulatory aberrations interfere with p53 network activity and influence response to platinum therapy (e.g., p53 negative regulators MDM2, MDM4 and WIP1) [139,186,219]. The E3 ubiquitin ligase MDM2 directly binds to p53 and promotes its nuclear export and proteasomal degradation, thus suppressing p53’s transcriptional activity [222]. In NEN, Akt activation and DAXX mutation may influence the stability of MDM2, regulating p53’s location, stability, and transcriptional activity, and sustaining proliferation or tumorigenesis [219,223,224]. In neuroendocrine prostate cancer, MDM2 amplification was related to the major effectiveness of platinum-based chemotherapy [186]. MDM2 can be inactivated after cisplatin-induced DNA damage, thus stabilizing the p53 protein and enabling it to induce cell cycle arrest and apoptosis [219,221].

Alterations of Rb (retinoblastoma protein), which are closely related to p53 mutations in NECs (Table S1) [33,38,140,183], were associated with better prognosis and response to platinum drugs-based therapy, especially in high-grade NENs (Figure 4; Table 1) [20,182,183,186,190,191]. The absence of Rb seemed to be more frequent in G3 NECs compared to G3 NETs and in SCNEC compared to LCNEC (Table S1) [182,191]. Derks and colleagues observed that patients with LCNEC tumors that carry a wild-type RB1 gene or express the Rb protein have a more favorable outcome when treated with platinum plus gemcitabine or taxanes compared to standard platinum plus etoposide chemotherapy, whereas no differences were observed when RB1 was mutated or the Rb protein not expressed [33]. However, in general, chemotherapeutic outcomes were better in tumors with abnormal Rb expression, sometimes associated with other genomic aberrations, such as KRAS [182,183], p16 [191], and MDM2 [186] (Table 1).

The overexpression of p16, a tumor suppressor that inhibits the CDK4/6 cell cycle regulators, can indicate a disruption of the Rb pathway. Several studies have confirmed an inverse relationship between the expression of Rb and p16 proteins in high-grade lung NETs. SCLC usually shows moderate or strong p16 staining in about 90% of the neoplastic cells [225]. Lacombe at al. observed a significantly higher response rate in NEC presenting high p16 levels, together with Rb loss (Table 1) [191].

A significant response to platinum therapy was observed in patients with advanced neuroendocrine prostate cancer who exhibited BRCA (breast cancer susceptibility gene) mutations [177,186,192,193,194,226]. BRCA1 and BRCA2 are important DNA repair genes that act as tumor suppressors. Their loss prevents DNA repair, leading to cell death after the formation of cisplatin DNA cross-links [226]. The positive association between BRCA2 mutations and response to platinum-based chemotherapy was clearly demonstrated in patients with prostate NEC. Both germline [192] and somatic [177,192,193] mutations of BRCA2 were found in these patients and were correlated with a high response to cisplatin/carboplatin and etoposide. Wood et al. also found somatic mutations in BRCA1 and BAP1 (BRCA1-associated protein) in colon LCNEC. Treatment with platinum-based therapy resulted in a complete response of the metastases, with no evidence of recurrence after 6.5 years. This led to the hypothesis that the loss or mutation of BRCA1 and/or BAP1 can predict response to platinum-based therapy, confirming the important role of genes involved in DNA repair in response to platinum drugs (Table 1) [195].

### 4.5. Other Genomic Alterations

Other mutations have recently been linked to the efficacy of chemotherapy in NENs. For instance, the speckle-type POZ protein, SPOP, is a zing finger protein with an oncogenic role that is frequently mutated in prostate and endometrial cancers [227,228]. It promotes the ubiquitination and degradation of proteins involved in tumor progression, such as PTEN and DAXX, thereby facilitating proliferation and inhibiting apoptosis in cancer cells [229]. Watanabe and colleagues showed that SPOP mutations in enzalutamide-resistant prostate cancer with NE differentiation improved treatment with platinum drugs (Table 1) [227].

The involvement of Wnt/β-catenin signaling in cancer was thoroughly described, and the altered expression of its components has also been observed in NETs [230]. Furukawa et al. demonstrated that β-catenin may serve as a reliable predictive biomarker for response to platinum-based chemotherapy in pancreatobiliary NEC [196], as evidenced by the increased expression of β-catenin in NET tissues and its correlation with tumor severity [231] (Table 1).

## 5. Influence of Specific Cellular Pathways in the Response to Platinum-Based Therapy in NENs

Bcl-2 is also closely related to NEN differentiation, as Bcl-2 expression is closely linked to chromogranin A (CgA) positivity, and tumor progression [232]. The Bcl system includes oncoproteins that affect apoptosis (such as Bax, Bad, Bid, and Bak) and proliferation (such as Bcl-2, Bcl-xL, and Raf), making it a key factor in regulating these processes [141]. Chemotherapeutic agents, including platinum drugs, can exert their cytotoxic effects by inducing intrinsic apoptosis through the mitochondrial pathway by modulating Bcl-2/Bax levels (Figure 3). The overexpression of Bcl-2 and reduction in Bax levels have been linked to resistance to platinum drugs in several cancers, including NENs [140,141,142,232,233]. In SCLC patients, Bcl-2 has been found to be overexpressed [234] and related to increased resistance to platinum chemotherapy in vitro [235], but its role in patients with NENs undergoing platinum-based chemotherapy has not yet been established.

Genomic analysis has revealed that NENs show alterations in chromatin remodeling genes, such as MEN1, DAXX (death domain-associated protein), and ATRX (α thalassemia/mental retardation syndrome X-linked) [236]. DAXX and ATRX cooperate with other genes in chromatin remodeling complexes. DAXX can also modulate the distribution of PTEN between the nucleus and the cytoplasm (Figure 4) [229,237]. Menin, encoded by MEN1, is involved in the regulation of DAXX and SMADs, ref. [238] and its loss of function has been associated with impaired DNA repair capability in NETs (Figure 4) [218]. Although Menin acts as a tumor suppressor in endocrine tissues, recent studies have shown that it can also promote tumorigenesis in various tumors. In some neoplasms, MEN1 has been found to act as a hub gene, interacting with and modulating several pathways. For example, Menin can inhibit the PI3K/Akt/mTOR pathway (by binding to Akt and preventing its translocation to the plasma membrane) [239,240] and the RAS-RAF-MEK1/2-ERK1/2 signaling pathway. Cherif et al. studied the in vitro response of prostate cancer PC-3 cells (which are positive for NE markers) to cisplatin treatment after Menin inhibition. They found that Menin activates the PI3K/Akt signaling pathway, which is associated with platinum drug resistance, and that Menin inhibition enhanced cisplatin sensitivity (by 69%) in PC-3 cells [240]. The role of MEN1 and DAXX/ATRX in the effectiveness of platinum-based treatments has not yet been defined.

Finally, other pathways have been linked to the differentiation and progression of NENs, but their involvement in cisplatin/carboplatin therapy has not yet been considered. Among these, the SMAD signaling pathway operates downstream of TGF-β and BMP ligands, regulating a diverse set of biological processes including proliferation, differentiation, and apoptosis [241,242]. Murai et al. were the first to show that in small-cell lung cancer (SCLC), TGF-β inhibited proliferation in vivo and tumor formation in vitro through the TGF-β-SMAD-ASCL1 pathway [243]. The loss of the tumor suppressor SMAD4 occurs in numerous solid organ neoplasms, included NENs, and it is associated with poor prognosis (Table S1) [143,144,145].

## 6. Conclusions

This review stems from the need to better understand the predictive and prognostic features of NENs. These features are still uncertain, and it is crucial to identify the major molecular genetic alterations in each type of tumor. Establishing correlations between specific abnormalities involved in tumorigenesis and the metastatic process could provide potential targets for cancer therapy. Due to the heterogeneity of NENs, there is a significant need to re-evaluate chemotherapeutic approaches, with a focus on studying combined and personalized therapies that can offer greater selectivity and effectiveness. The review incorporates recent and relevant studies in the field, providing a comprehensive analysis of current knowledge and developments in NENs and platinum-based chemotherapy responses, taking into account data and research findings up to the year 2024.

Among the available therapeutic options, platinum-based chemotherapy is considered the first-line treatment for well-differentiated G3 NETs and NECs, particularly when the Ki-67 index is higher than 55% or in cases of rapid clinical progression. The cellular response to platinum-based chemotherapy is a complex process that typically begins with the induction of DNA damage, followed by a series of events involving signal transduction and the activation of transcription factors. These factors induce the expression of numerous genes involved in various cellular functions, such as DNA repair, cell cycle arrest, cell death, and the inhibition of epithelial–mesenchymal transition (EMT) (Figure 3). Additionally, there may be crosstalk between different signaling pathways, resulting in diverse downstream effects. Also, with this review, we underscore the pivotal role of molecular genetic alterations in NENs and their impact on treatment outcomes. It is very important to identify the major molecular genetic alterations specific to each tumor type, essential for advancing personalized medicine in NENs. Current guidelines recommend using MEN1, DAXX/ATRX, and RB1/TP53 to distinguish between P-NET G3 and P-NEC. The distinct genetic abnormalities between P-NETs (G3) and P-NEC may explain why P-NETs have a lower sensitivity to platinum-based chemotherapy compared to P-NECs. Consistently, NECs are often characterized by the aberrant expression of p53 and/or Rb proteins, as well as KRAS mutations (Table S1), which have been linked to a higher response to platinum-based chemotherapy (Figure 4; Table 1). On the other hand, diagnosing LCNEC can be challenging due to its histological similarities with non-small-cell lung cancer (NSCLC) and, in some cases, small-cell lung cancer (SCLC) (as evidenced by the PI3K/Akt/mTOR pathway and other gene alterations) (Table S1). As platinum-based chemotherapy is the standard treatment for SCLC, it is often used for both LCNEC and NSCLC. Interestingly, although platinum-based chemotherapy is not typically used for neuroendocrine prostate cancer, it has been reported to be effective in some cases. This is likely due to the presence of BRCA2, PTEN, and MDM2 mutations, which make this type of cancer responsive to platinum-based chemotherapy (Table 1).

The relationship between specific genetic abnormalities and the processes of tumorigenesis and metastasis presents a promising area for future research. Understanding these correlations could lead to the identification of novel targets for cancer therapy, thereby enhancing treatment efficacy. The heterogeneity of NENs suggests that personalized treatments, based on the genetic characteristics of each tumor, could offer better selectivity and effectiveness. The prospects for improved treatment outcomes through personalized and combined therapeutic approaches represent a significant advancement in the field of neuroendocrine neoplasms.

## Figures and Tables

**Table 1:** Genomic alterations and aberrant regulations impacting response to platinum-based chemotherapy in neuroendocrine neoplasms (NENs). The utilization of IHC (immunohistochemistry) staining, NGS (next-generation sequencing), PCR (polymerase chain reaction), CGP (comprehensive genomic profiling) techniques for the identification of therapeutic targets and deregulated pathways with positive (green) or negative (red) influence on chemotherapy response. Yellow represents members with uncorrelated effects on chemotherapy.

Altered Gene/ Biomarker	NE Tumor Type	Type of Analyses	Observations	References
**MAPK Pathway**				
**KRAS**	G3 P-NET (n = 21) P-NEC (n = 31 SCNEC; n = 18 LCNEC)	PCR IHC	KRAS mutations are not detected in NET-G3 (0%), while NEC-G3 harbors KRAS mutations in 48.7% of cases. There are no significant differences between SCNEC and LCNEC in the prevalence of KRAS mutations. KRAS mutations are associated with a higher response to platinum-based chemotherapy compared to those without mutations (mutated KRAS, 77% vs. wild-type, 23%).	[182]
	G3 P-NET (n = 21) P-NEC (n = 18 LCNEC; n = 31 SCNEC)	IHC Real-Time PCR	KRAS is mutated in 48.7% of G3 P-NEC. Patients with a KRAS mutation exhibit a better response to first-line platinum-based therapy compared to those with wild-type KRAS but tend to have shorter overall survival rates.	[183]
	G3 NET (n = 6) NEC (n = 77)	Real-Time PCR NGS	KRAS mutations do not affect treatment effectiveness or survival rates following initial chemotherapy.	[184]
**BRAF**	G3 NET (n = 6) NEC (n = 77)	Real-Time PCR NGS	A higher frequency of BRAF mutations is found in colon NEC and predicts failure to first-line treatment with cisplatin/carboplatin and etoposide.	[184]
**PTEN/PI3K/Akt/mTOR Pathway**				
**PTEN**	prostate NEC (n = 1)	NGS IHC	Somatic mutations in PTEN (and BRCA2) were identified in the tumor tissue. The tumor cells exhibited decreased staining for PTEN, indicating a loss of protein expression, which is also associated with a significant response to platinum therapy.	[177]
**Notch/ASCL1 pathway**				
**Notch1**	SCLC (n = 46)	IHC	Hes1, ASCL1, and DLL3 protein expression levels are not associated with sensitivity to platinum chemotherapy or prognosis. However, SCLC with low Notch-1 expression has a better survival rate.	[131]
**DLL3**	LCNEC (n = 70)	IHC	DLL3 is a predictive marker for sensitivity to platinum-based adjuvant chemotherapy in LCNEC. Patients with DLL3-negative tumors who receive chemotherapy show significantly higher overall survival and recurrence-free survival rates.	[185]
**Members of pathways involved in DNA repair**				
**MDM2**	prostate NEC (n = 1)	NGS	Platinum-based chemotherapy was found to be effective in a patient with pancreatic neuroendocrine carcinoma (NEC) exhibiting an aggressive course and MDM2 amplification.	[186]
**p53**	NSCLC-NE (n = 157)	IHC	There is no statistically significant correlation between the p53 marker and response to chemotherapy. However, patients with an increased expression of p53 are more likely to experience progressive disease after undergoing chemotherapy.	[132]
	G3 NET (n = 10) LCNEC (n = 31) SCNEC (n = 48)	IHC	There is no statistically significant correlation between the p53 marker and response to chemotherapy. However, patients with an increased expression of p53 are more likely to experience progressive disease after undergoing chemotherapy.	[132]
	ES-SCLC (n = 75)	NGS	Patients with mutant TP53 had a better PFS than those with wild-type TP53.	[187]
	P-NET (n = 50) P-NEC (n = 29)	IHC	Abnormal p53 expression is not associated with response to platinum-based therapy.	[20]
	Prostate NEC (n = 1)	NGS IHC	The TP53 p.P72R variant is correlated with higher platinum sensitivity and longer survival of patient with aggressive prostate cancer.	[188]
	G3 GEP-NET (n = 41) GEP-NEC (n = 188)	NGS	TP53 mutation predicts an inferior response rate to cisplatin/carboplatin for NEC but does not correlate with overall survival (except for small-cell NEC).	[189]
**Rb**	SCLC (n = 50)	Whole/Targeted Genome Sequencing IHC Western Blotting	The RB1 mutation status had the most significant impact of any gene. SCLC patients with wild-type RB1 demonstrated a significantly lower response to chemotherapy compared to patients with mutant RB1.	[190]
	G3 P-NET (n = 21) P-NEC (n = 31 SCNEC; n = 18 LCNEC)	PCR IHC	The loss of Rb expression was not observed in NET-G3 (0%), while NEC-G3 showed a loss of expression in 54.5% of cases. There were no significant differences in the prevalence of abnormal Rb expression between SCNEC and LCNEC. The loss of Rb in NECs was associated with a significantly higher response rate to platinum-based chemotherapy compared to those without (80% vs. 24% with normal Rb expression).	[182]
	G3 P-NET (n = 21) P-NEC (n = 18 LCNEC; n = 31 SCNEC)	IHC Real-Time PCR	The rate of Rb loss in G3 P-NEC is 54.5% and is associated with a higher response rate to first-line platinum-based regimens compared to those without Rb loss. However, patients with Rb loss tended to have shorter overall survival rates than those without Rb loss.	[183]
	prostate NEC (n = 1)	NGS	A patient with heterozygosity loss in the RB1 gene displayed an aggressive course and responded favorably to chemotherapy containing platinum.	[186]
	G3 NET (n = 10) LCNEC (n = 31) SCNEC (n = 48)	IHC	Patients with G3 neuroendocrine neoplasms (NENs) exhibit varying responses to treatment with etoposide and platinum. However, the objective response rate was notably higher in NENs lacking the retinoblastoma (Rb) gene (63% vs. 42%).	[191]
**BRCA**	prostate NEC with metastatic lung nodule and brain metastases (n = 1)	NGS	Combined platinum and etoposide chemotherapy yields partial and complete remissions of brain and lung metastases, respectively, in a patient with a somatic and germline BRCA2 mutation.	[192]
	prostate NEC (n = 1)	NGS	A patient with a complete copy number loss of BRCA2 and ATM in prostate NEC (but not in his original adenocarcinoma) exhibited a complete response to carboplatin plus etoposide chemotherapy.	[193]
	prostate NEC (n = 1)	NGS	A patient with a BRCA2 mutation (along with a PTEN mutation) displays an aggressive disease progression and showed a positive response to chemotherapy containing platinum.	[186]
	prostate SCNEC (n = 1)	PCR	The patient with a germline BRCA2 mutation achieved a complete response to platinum-based chemotherapy but experienced a limited duration of remission when treated with olaparib (a PARP inhibitor) as maintenance therapy.	[194]
	colon LCNEC (n = 1)	CGP	Treatment with platinum-based therapy leads to a full radiographic remission of the metastases, with no indication of recurrence after 6.5 years. The response to the therapy is probably attributed to the loss of BRCA1 and/or BAP1 function.	[195]
	prostate NEC (n = 1)	NGC IHC	BRCA2 is mutated in tumors but not in normal tissue. BRCA2 somatic mutations are associated with a strong response to platinum therapy.	[177]
**Other Markers**				
**β-catenin**	pancreatobiliary NEC (n = 30)	IHC	Higher levels of β-catenin are a predictive factor for response to platinum-based chemotherapy.	[196]
**p16**	G3 NET (n = 10) LCNEC (n = 31) SCNEC (n = 48)	IHC	The objective response rate is significantly higher in NEN with high p16 levels (66% vs. 35%).	[191]

## Supplementary Materials

The following supporting information can be downloaded at: https://www.mdpi.com/article/10.3390/ijms25168568/s1.

## Abbreviations

5-FU: 5-Fluorouracil; ABC: ATP-binding cassette; AC: atypical carcinoid; AFP: alpha-fetoprotein; Akt/PKB: protein kinase B; AMPK: AMP-activated protein kinase; ASCL1: achaete-scute homolog 1; ASCO: American Society of Clinical Oncology; ATP: adenosine triphosphate; ATPase: adenosine triphosphatases; ATR: ataxia-telangiectasia-mutated and Rad 3-related; ATRX: X-linked mental retardation and α-thalassaemia syndrome protein; BAP1: BRCA1-associated protein; BCL-2: B-cell lymphoma 2; BMP: bone morphogenetic protein; BP: bronchopulmonary; BRCA1/2: breast cancer gene 1/2; CAPOX: capecitabine plus oxaliplatin; CAPTEM: capecitabine plus temozolomide; CDK: cyclin-dependent kinase; CgA: chromogranin A; CGP: comprehensive genomic profiling; CSF1R: colony-stimulating factor receptor 1; CTLA-4: cytotoxic T-lymphocyte antigen 4; CTR: copper transporter; Cyt-C: cytochrome C; DAXX: death domain-associated protein; DLL3: delta-like protein 3; DNA: deoxyribonucleic acid; DNA-PK: DNA-dependent protein kinase; DOXO: doxorubicin; EGF: epidermal growth factor; ERK: extracellular signal-regulated kinase; ES: extensive-stage; ESMO: European Society for Medical Oncology; FGF: fibroblast growth factor; FLT3: Fms-related tyrosine kinase 3; FOLFOX: fluorouracil plus oxaliplatin; GEP: gastroenteropancreatic; GI: gastrointestinal; GSH: glutathione; HCG: human chorionic gonadotropin; HIF: hypoxia-inducible factor; HR: homologous recombination; ICI: immune checkpoint inhibitor; IGF: insulin-like growth factor; IHC: immunohistochemistry; INSM-1: insulinoma-associated protein-1; Isl-1: Islet 1; JNK: c-Jun N-terminal kinase; KIT: stem-cell growth factor receptor; LAR: long-acting release; LCNEC: large-cell neuroendocrine carcinoma; LRRC: leucine-rich repeat containing; MAPK: mitogen-activated protein kinase; MATE: multidrug extrusion transporter; MCC: Merkel cell carcinoma; MDM: murine double minute; MEN: multiple endocrine neoplasia; MTC: medullary thyroid carcinoma; mTOR: mammalian target of rapamycin; NE: neuroendocrine; NEC: neuroendocrine cancer; NEN: neuroendocrine neoplasm; NER: nucleotide excision repair; NET: neuroendocrine tumor; NGS: next-generation sequencing; NHEJ: non-homologous end joining; Notch: neurogenic locus notch homolog; NSCLC: non-small-cell lung cancer; NSE: neuron-specific enolase; OCT: organic cation transporter; P: pancreatic; p-Akt: phosphorylate Akt; PARP: poly(ADP-ribose) polymerase; PAX 8: paired box 8; PCR: polymerase chain reaction; PD-1: programmed death-1; PDGFR: platelet-derived growth factor; PD-L1: programmed death-1 ligand; PI3K: phosphatidylinositol 3-kinase; p-mTOR: phosforilate mTOR; PP: pancreatic polypeptide; PRRT: peptide receptor radionuclide therapy; PTEN: phosphatase and tensin homolog; Rb: retinoblastoma protein; RET: rearranged during transfection; ROS: reactive oxygen species; SCLC: small-cell lung carcinoma; SCNEC: small-cell neuroendocrine carcinoma; SMAD: small mother against decapentaplegic; SPOP: speckle-type POZ protein; SSA: somatostatin analog; SSTR: somatostatin receptor; STZ: streptozotocin; SYN: synaptophysin; TC: typical carcinoid; TGF-α/β: transforming growth factor-α/β; TGF-βR1,2: transforming growth factor-β receptor; TKI: tyrosine kinase receptor inhibitors; TTF-1: thyroid transcription factor-1; VEGF: vascular endothelial growth factor; VEGFR: vascular endothelial growth factor receptor; VHL: von Hippel–Lindau syndrome; VIP: vasoactive intestinal peptide; VRAC: volume-regulated anion channel; WHO: World Health Organization; WIP1: wild-type p53-induced phosphatase 1; Wnt: wingless-related integration site.
