# Supplementary material for: An Overview of Altered Pathways Associated with Sensitivity to Platinum-Based Chemotherapy in Neuroendocrine Tumors: Strengths and Prospects
Source: Int J Mol Sci. 2024 Aug 6;25(16):8568. doi: 10.3390/ijms25168568 (PMC11354135; doi:10.3390/ijms25168568)
Supplement: Supplementary file 1 [file ijms-25-08568-s001.zip › ijms-3060012-supplementary.pdf]

Supplementary Materials

Table S1. Genomic alterations and aberrant regulations impacting response to platinum-based chemotherapy in Neuroendocrine Neoplasms (NENs). Utilization of IHC (Immunohistochemistry) staining, NGS (Next-Generation Sequencing), PCR (Polymerase Chain Reaction), CGP (Comprehensive Genomic Profiling) techniques for identification of therapeutic targets and deregulated pathways with positive (green) or negative (red) influence on chemotherapy response. Yellow represents members with uncorrelated effects on chemotherapy.

Signaling Pathway	Tumor type	Observations	References
**MEN1**	P-NET	The MEN1 gene is mutated in 44% of pancreatic NETs (P-NETs)	[135]
	P-NET	79% of P-NETs have germline MEN1 mutations.	[146]
	P-NET (G3)<br>P-NEC<br>MiNEC	The MEN1 gene is mutated in 71% of G3 P-NETs.	[137]
	P-NET	Biallelic loss of MEN1, caused by deleterious mutations, copy number losses, and/or loss of heterozygosity events, was identified in four out of five cases. Additionally, low mRNA expression of MEN1 was observed in all five cases.	[136]
**DAXX/ATRX**	P-NET	43% of P-NETs have a mutation in the DAXX/ATRX pathway.	[135]
	SCNEC<br>LCNEC<br>P-NET	DAXX and ATRX are absent in 45% of P-NETs, but present in SCNEC and LCNEC.	[140]
	P-NET	The loss of DAXX or ATRX is correlated with chromosomal instability in P-NETs and reduced survival rates in patients.	[147]
	P-NET	Mutations in DAXX are more common in larger P-NETs and are linked to characteristics of malignancy (grade, nodal involvement, and lymphovascular invasion).	[148]
	P-NET (G3)<br>P-NEC<br>MiNEC	The G3 P-NETs exhibit frequent mutations in DAXX (47%).	[137]
	P-NET	Biallelic loss of DAXX, caused by deleterious mutations, copy number losses, and/or loss of heterozygosity events, was identified in four out of five cases. Additionally, low mRNA expression of DAXX was observed in all five cases.	[136]
**Notch/ASCL1 pathway**	P-NET<br>P-NEC	There is no correlation between the expression of Notch-1 and clinical data such as tumor size, malignancy, metastases, or survival.	[149]
	MCC	Notch-1 is expressed in Merkel cell carcinoma (MCC) and could potentially serve as a diagnostic tool for cutaneous neuroendocrine tumors. No suppressor function of Notch-1 has been identified in MCC.	[150]
	GI-NET	ASCL1 is specifically upregulated in a majority of gastrointestinal (GI) tumors. The Notch signaling pathway plays a significant role in regulating neuroendocrine differentiation and serotonin production in GI carcinoid tumors.	[134]
	SCLC	High expression of Notch1 is associated with a favorable prognosis.	[151]
	SCLC<br>LCNEC	SCLCs and a subset of LCNECs demonstrate elevated levels of ASCL1 and DLL3, and reduced levels of Notch. Another subset of LCNECs displays alterations in TP53 and RB1 and differs from the majority of SCLC tumors (with low levels of ASCL1 and DLL3, and high levels of Notch).	[152]
	SCLC	83% of patients have high DLL3 expression, while a minority has high Notch1 expression (22%). Notch1 and ASCL1 expressions are negatively correlated. Low expression of Notch1 is associated with a better prognosis.	[131]
**p53 / Rb1**	GEP-NEC	Alterations in the p53 gene are not associated with the development and progression of gastroenteropancreatic neuroendocrine carcinomas (GEP-NECs).	[153]
	GI-NEC<br>GI carcinoid	Classical carcinoids do not exhibit nuclear p53 expression, whereas all carcinoid-like NECs demonstrate expression of mutant p53 protein.	[154]
	AC<br>TC<br>SCLC<br>LCNEC	TC and AC are genetically distinct from SCLC and LCNEC. p53 mutations are linked to a more malignant phenotype in lung NET.	[155]

	AC<br>TC<br>LCNEC	Mutations in the p53 gene are rare in these pulmonary NETs and are linked to tumors of higher grade.	[138]
	NSCLC	The normal expression of p53 in NSCLC-NE is a favorable factor for both survival and time to disease progression.	[132]
	P-NET	MDM2, MDM4, and WIP1 are upregulated in P-NET and suppress p53, resulting in tumor initiation and progression.	[139]
	SCLC<br>BP carcinoid<br>GI carcinoid	Copy number alterations of the RB1 tumor suppressor gene are common in small cell lung cancer (SCLC), bronchial carcinoids, and carcinoids of gastrointestinal (GI) origin.	[156]
	SCNEC<br>LCNEC<br>P-NET	Abnormal immunolabeling patterns of p53 (95%) and Rb (74%) are common in both small cell neuroendocrine carcinomas (SCNECs) and large cell neuroendocrine carcinomas (LCNECs) and are associated with intragenic mutations in the TP53 and RB1 genes. Immunolabeling of p53 and Rb is normal in pancreatic neuroendocrine tumors (P-NETs).	[140]
	NEC	The Rb protein is lost in 90% of prostatic small cell neuroendocrine carcinomas.	[157]
	SCLC	The loss of TP53 and Rb1 (91-98%) appears to be mandatory in SCLC.	[38]
	GI-NET<br>GI-NEC	The frequency of aberrant p53 expression and loss of Rb is significantly higher in NECs than in NETs. In patients with GI-NEC, the percentage of aberrant p53 mutations is higher in LCNEC compared to SCNEC, while loss of Rb is more prevalent in SCNEC.	[158]
	LCNEC	TP53 and RB1 are frequently mutated genes in LCNEC, with a prevalence of 78% and 38%, respectively.	[159]
	NET<br>NEC	TP53 mutations are found in 57% of poorly differentiated NECs and are correlated with a worse survival rate.	[37]
	LCNEC	TP53 is mutated in 85% of large cell neuroendocrine carcinomas (LCNECs), while RB1 is mutated in 47%. RB1 was co-altered with TP53 in 92% of the tumors with RB1 mutations.	[33]
	SCLC<br>LCNEC	Inactivating mutations in TP53 and RB1 are present in LCNEC (71% and 26%, respectively). The frequency of mutations in RB1 is lower than that in SCLCs (40%).	[160]
	GI-NET<br>GI-NEC	Dysregulation of the Rb/E2F pathway (92% of cases) is essential in the pathogenesis of colorectal NEC.	[161]
	SCNEC	Mutations in the TP53 gene are rare in cervical NEC (13%).	[162]
	GI-NET	Abnormal expression of p53 and Rb is associated with various subtypes of gastric NEC, resulting in an increased number of lymph node metastases and a higher tumor mutation burden stage.	[163]
**Bcl system**	AC<br>TC<br>SCLC<br>LCNEC	Bcl-2 is highly expressed in approximately 90% of patients with LCNEC and SCLC, in contrast to patients with typical carcinoid (18.7%) and atypical carcinoid (0%). Overexpression of Bcl-2 and Bcl-2/Bax ratios are linked to a p53 mutant immunophenotype.	[142]
	SCLC	The expression of Bcl-2 and Bax decreases in SCLC with metastases, indicating a decrease in apoptosis and the development of metastatic disease.	[141]
	SCNEC<br>LCNEC<br>P-NET	The Bcl-2 protein is overexpressed in 100% of small cell neuroendocrine carcinomas (SCNEC) and in 50% of large cell neuroendocrine carcinomas (LCNEC), compared to only 18% of pancreatic neuroendocrine tumors (P-NETs).	[140]
	GI-NET<br>GI-NEC	The expression of Bcl-2 is significantly higher in NECs (64%) compared to NETs (5%) and poorly differentiated adenocarcinomas (5%). In patients with GI-NEC, Bcl-2 expression is significantly higher in LCNEC compared to SCNEC.	[158]
**MAPK pathway**	AC<br>TC<br>SCLC<br>LCNEC	Analysis of KRAS-2 and c-raf gene sequences revealed no indication of point mutational alterations in the examined tumors.	[155]
	AC<br>TC<br>LCNEC	KRAS mutations appear to be correlated with higher-grade carcinomas. No N-RAS or H-RAS mutations were detected in these pulmonary NETs.	[138]
	GEP-NET	BRAF mutations do not play a role in the tumorigenesis of GEP-NETs. However, activation of the RAF/MAPK pathway may have a causative role in the development of GEP-NETs, regardless of BRAF or KRAS-2 mutation.	[164]
	AC<br>TC<br>SCLC<br>NSCLC-NE	70% of non-small cell lung cancers (NSCLCs) exhibit positivity for p-ERK1/2. In contrast, significantly lower rates of positivity are observed in other lung tumors known for neuroendocrine (NE) differentiation (0% for typical carcinoid, 20% for atypical carcinoid, and 40% for small cell lung cancer [SCLC]). This suggests that the	[133]

		ERK/MAPK signal transduction pathway may play a role in the NE differentiation of NSCLC.	
	Colorectum cancer (with or without NE differentiation)	KRAS is mutated in 50% of tumors, while BRAF and NRAS are mutated in 11% and 5% of tumors, respectively. KRAS mutations are not significantly associated with NE differentiation.	[165]
	LCNEC	BRAF V600E mutations were found in 62.5% of colon LCNECs.	[166]
	GI-NET	P38 is highly expressed in small intestinal NETs and phosphorylated ERK is upregulated in liver metastases.	[167]
**PI3K/Akt/mTOR pathway**	Cervixal NEC	67% of cervical NECs have PI3K or MAPK pathway activating mutations. Mutations in the catalytic subunit of phosphatidylinositol 3-kinase (PIK3CA) are the most frequently observed oncogenic mutation (27%).	[162]
	GEP-NET GEP -NEC	The expression of p-mTOR is higher in poorly-differentiated NECs (67%) compared to well-differentiated NETs and NECs (27%).	[168]
	NEC	mTOR is expressed in 80% of patients with poorly differentiated NEC. There is no correlation between mTOR expression and tumor origin (pancreas, colon, lung, small bowel, and others) or proliferation rate.	[169]
	P-NET	The mTOR gene is mutated in 15% of P-NET, compared to PDAC (0.8%).	[135]
	GEP-NET	The expression and activity of mTOR were elevated in GEP-NETs (greater in foregut than in midgut tumors). In foregut tumors, increased mTOR expression is associated with a higher proliferative capacity and the presence of distant metastases.	[170]
	P-NET	mTOR is overexpressed in well-differentiated P-NET patients, with a higher rate of mTOR activation in G2 compared to G1 P-NETs. There is no significant correlation between mTOR activation and patients' prognosis.	[171]
	GEP-NET TC (G1)	Enhanced p-Akt expression and PTEN deficiency are associated with the metastatic capacity and aggressive behavior of low-grade neuroendocrine tumors (NETs).	[130]
	SI-NET	Amplification of Akt1 or Akt2 is the most prevalent occurrence in patients with modifications of PI3K/Akt/mTOR signaling.	[145]
	P-NET	PTEN mutations are present in a minority (3%) of P-NETs.	[128]
	P-NET	PTEN is downregulated in the majority of P-NETs; its decreased expression is linked to a shorter period of being disease-free and overall survival.	[129]
	NEC	The observation shows that 63% of small cell neuroendocrine carcinomas exhibit a loss of PTEN protein.	[157]
	SCLC	PTEN is mutated in 9% of SCLC cases and is associated with increased proliferation and cell survival.	[38]
	GEP-NET TC (G1)	Lung and appendiceal low-grade NETs consistently exhibit elevated PTEN expression and a more favorable prognosis compared to their gastroenteropancreatic (GEP) counterparts. The absence of PTEN was found to be associated with increased p-Akt expression and a more aggressive phenotype in low-grade NETs.	[130]
**SMAD signaling**	GI-NET	46% of patients with small intestine NETs have mutated or deleted SMAD genes. The most common event is the deletion of SMAD2 and SMAD4, while one patient has a mutation in SMAD1.	[145]
	GI-NET	The absence of SMAD4 in primary GI-NETs tumors is negatively associated with post-surgical outcomes.	[143]
	GI-NET	The loss of SMAD4 is a frequent occurrence in small intestine NETs and is associated with metastatic disease.	[144]
